# Genome-wide identification, phylogenetic and expression analysis of the heat shock transcription factor family in bread wheat (*Triticum aestivum* L.)

**DOI:** 10.1186/s12864-019-5876-x

**Published:** 2019-06-18

**Authors:** Min Zhou, Shigang Zheng, Rong Liu, Jing Lu, Lu Lu, Chihong Zhang, Zehou Liu, Congpei Luo, Lei Zhang, Levi Yant, Yu Wu

**Affiliations:** 1 Chengdu Institute of Biology, Chinese Academy of Sciences, No.9, section 4 of South RenMin Road, Wuhou District, Chengdu, 610041 Sichuan China; 2 University of Chinese Academy of Sciences, Beijing, 100049 China; 3 School of Life Sciences, University of Nottingham, Nottingham, NG7 2RD UK

**Keywords:** Wheat, *Hsf*, Genome-wide analysis, Expression profiles

## Abstract

**Background:**

Environmental toxicity from non-essential heavy metals such as cadmium (Cd), which is released from human activities and other environmental causes, is rapidly increasing. Wheat can accumulate high levels of Cd in edible tissues, which poses a major hazard to human health. It has been reported that heat shock transcription factor A 4a (*HsfA4a*) of wheat and rice conferred Cd tolerance by upregulating metallothionein gene expression. However, genome-wide identification, classification, and comparative analysis of the *Hsf* family in wheat is lacking. Further, because of the promising role of *Hsf* genes in Cd tolerance, there is need for an understanding of the expression of this family and their functions on wheat under Cd stress. Therefore, here we identify the wheat *TaHsf* family and to begin to understand the molecular mechanisms mediated by the *Hsf* family under Cd stress.

**Results:**

We first identified 78 putative *Hsf* homologs using the latest available wheat genome information, of which 38 belonged to class A, 16 to class B and 24 to class C subfamily. Then, we determined chromosome localizations, gene structures, conserved protein motifs, and phylogenetic relationships of these *TaHsfs*. Using RNA sequencing data over the course of development, we surveyed expression profiles of these *TaHsfs* during development and under different abiotic stresses to characterise the regulatory network of this family. Finally, we selected 13 *TaHsf* genes for expression level verification under Cd stress using qRT-PCR.

**Conclusions:**

To our knowledge, this is the first report of the genome organization, evolutionary features and expression profiles of the wheat *Hsf* gene family. This work therefore lays the foundation for targeted functional analysis of wheat *Hsf* genes, and contributes to a better understanding of the roles and regulatory mechanism of wheat *Hsfs* under Cd stress.

**Electronic supplementary material:**

The online version of this article (10.1186/s12864-019-5876-x) contains supplementary material, which is available to authorized users.

## Background

Heat shock proteins (HSPs) perform important roles not only in cellular protection against stress-related damage, but also in the regular folding, intracellular distribution, and degradation of proteins. These functions facilitate organismal survival under stressful conditions [1, 2]. Heat shock transcription factors (*Hsfs*) modulate the expression of HSPs, and participate in various aspects of protein homeostasis, such as refolding, assembly and transporting damaged proteins, which sustain protein stability [3–7]. *Hsfs* share a core structure consisting of an N-terminal DNA binding domain (DBD) and an adjacent bipartite oligomerization domain (HR-A/B) [6, 8]. Some *Hsfs* also share a leucine-rich nuclear export signal (NES) for nuclear export, a nuclear localization signal (NLS) essential for nuclear import,, and short peptide motifs (AHA motifs) for activator functions [9–12]. Based on the characteristics of their HR-A/B domain and phylogenetic comparisons, plant Hsf genes may be classified into three broad groups: A, B and C [6, 8]. The HR-A/B regions of class B *Hsfs* are relatively compact, not including any insertions, while all class A and class C HSFs have an outspread HR-A/B region due to an insertion of 21 (class A) and seven (class C) amino acid residues [6]. This classification is also supported by differences in the flexible linkers between the DBD domain and HR-A/B domain, which consists of 9 to 39, 50 to 78, and 14 to 49 amino acid residues in class A, B and C *Hsfs*, respectively [6, 9]. Recent studies indicate that *Hsfs* are engaged in plant development and growth, as well as in response to abiotic stresses such as salt, cold, drought and cadmium challenge [7, 9, 13–19]. For example, *HsfA9* is related to seed maturation and embryogenesis in sunflowers and Arabidopsis [14–16]. *HsfA4a* is involved in cadmium tolerance in wheat [19]. Due to the essential modulatory functions of *Hsf* genes in plants [16–18], the *Hsf* gene family has been studied in the models *Arabidopsis thaliana* and rice (*Oryza sativa*), and nonmodels such as poplar (*Popupus trichocarpa*), maize *(Zea mays*), and apple (*Malus domestica*) [5, 6, 9, 20–22]. However, the *Hsf* gene family in the bread wheat (*Triticum aestivum*) has not been systematically examined.

Bread wheat is one of the most widely grown and consumed crops worldwide [23]. Bread wheat is hexaploid (2n = 6x = 42; AABBDD genome), originating from two amphiploidization events: the first hybridization producing the tetraploid wheat species (2n = 4x = 28, genome AABB) was between the *Triticum urartu* (2n = 2x = 14, genome AA) and presumably *Aegilops speltoides*, belonging to the section Sitopsis (2n = 2x = 14, genome SS); the second hybridization was between the tetraploid wheat and *Aegilops tauschii* (2n = 2x = 14, genome DD) [24, 25]. Therefore, bread wheat has a huge and highly complex genome with three subgenomes (A, B and D) and ~17Gb total size [26], leading to great challenges for genomic studies. Recently, however, a quality draft genome of hexaploid ‘Chinese Spring’ wheat has provided the foundation upon which we can investigate wheat gene families and to clearly recognize homologous gene copies in these three sub-genomes [27]. Further, it has allowed the study of interactions of loci during polyploidization and the retention and dispersion of homologous gene [28, 29].

Here we first perform an in silico genome-wide study to comprehensively identify members of the wheat *Hsf* gene family. Next, to characterize evolutionary and functional features, we determine chromosome locations, gene structures, conserved protein domains, phylogenetic relationships and expression profiles for this family. Our study provides a foundation for downstream targeted functional investigation of wheat *Hsf* genes, and will be allow for better understanding of the molecular mechanisms by which *Hsfs* regulate in growth, development and stress resilience in wheat.

## Results

### Genome-wide identification and classification of *Hsf* family in wheat

Through the availability of the genome sequence, it is possible for the first time to identify all the *Hsf* family members in wheat. In this study, we identified a total of 78 genes as *Hsf* members in the wheat genome, designating the predicted wheat *Hsf* genes *TaHsf1* to *TaHsf78*. Members of the *Hsf* gene family have been broadly subdivided into Classes A, B, and C according to differences in the length of the flexible linkers between the A and B parts of the HR-A/B regions. In the T*aHsf* gene family, 38, 16 and 24 genes were accordingly assigned to Classes A, B and C, respectively. Within the A clade, 8 distinct subclades (A1, to A8) were resolved. The B-type *Hsf* genes were grouped into a separate clade subdivided into three groups (B1, B2 and B4). And the C-type genes were subdivided into two groups (C1 and C2). We further performed a BLASTN search against the wheat expressed sequence tag (EST) using the 78 identified *Hsfs* as queries to verify the existence and completeness of this set of wheat *Hsfs*. Results showed that most of the T*aHsfs* were supported by EST hits except 2 *Hsfs* (T*aHsf57* and *TaHsf75*). We speculated these 2 unsupportted *TaHsfs* might not be expressed under any the assayed conditions or may be expressed at very low level that cannot be easily detected. Among the supported *TaHsf* genes, *TaHsf8* has the largest number of EST hits, with 49, followed by *TaHsf21* and T*aHsf27* with 48 and 30 hists, respectively. Chromosome localization analysis found that 4 *TaHsfs* did not have corresponding chromosomal locations, and that the remaining 74 *TaHsf* genes were distributed on all of the 21 wheat chromosomes. Chromosome 3B contained the most *Hsf* genes with 8, followed by 4B, 5A and 5D, with each harboring 6, then 3A with 5, and finally 6A, 6B and 6D with one each. The predicted lengths of the putative TaHsf proteins ranged from 209 to 701 amino acids, with the molecular weights (Mw) ranging from 22.72 to 73.92 kDa and theoretical isoelectric points (PI) ranging from 4.67 to 9.50 (Table 1).

**Table 1:** The list of the putative wheat Hsf genes

Names	Ensemble Gene ID	Chromosome location	EST count	length (bp)	Exons	Introns	Amino acid length (aa)	PI	MW (kDa)
*TaHsf1*	Trae_4AL_8577C148B	scaffold_288809_4AL: 49,335-56,655	26	7321	3	2	521	4.94	57.34
*TaHsf2*	Trae_5BL_E15759DAD	scaffold_404129_5BL: 211,116-217,536	26	6421	2	1	471	5.18	52.89
*TaHsf3*	Trae_5DL_B1D24781B1	scaffold_433347_5DL: 108,916-114,305	28	5390	2	1	487	4.95	54.60
*TaHsf4*	Trae_5AL_16AD8DEEC	scaffold_375092_5AL: 45,746-49,544	4	3799	2	1	346	5.45	38.98
*TaHsf5*	Trae_5DL_6EB179C88	scaffold_434875_5DL: 17,703-21,445	8	3743	2	1	348	5.39	38.90
*TaHsf6*	nd	scaffold_640974_U: 63,006-67,190	6	4185	2	1	353	5.59	39.72
*TaHsf7*	Trae_2AS_CF07F4EC2	scaffold_113503_2AS: 55,860-61,955	26	6096	2	1	413	4.99	45.60
*TaHsf8*	Trae_2BS_ECF9B4EB4	scaffold_148328_2BS: 27,356-32,557	49	5202	2	1	405	5.06	44.92
*TaHsf9*	Trae_2DS_B6872CB84	scaffold_177319_2DS: 131,495-137,254	27	5760	2	1	412	4.85	45.43
*TaHsf10*	Trae_3AL_E15419B88	scaffold_194616_3AL: 22,466-26,620	2	4155	2	1	314	6.14	35.42
*TaHsf11*	TRAES3BF002300100CFD	scaffold_221589_3B: 97,736-102,700	4	4965	3	2	396	5.09	43.99
*TaHsf12*	nd	scaffold_379543_5AL: 6399-8897	10	2499	3	2	372	5.37	41.13
*TaHsf13*	nd	scaffold_433195_5DL: 110,106-113,560	9	3455	2	1	377	5.42	41.59
*TaHsf14*	nd	scaffold_116363_2AS: 3667-6615	7	2949	2	1	467	6.06	51.62
*TaHsf15*	Trae_2AS_53BFA14C7	scaffold_114504_2AS: 30,086-34,710	7	4625	4	3	502	5.95	55.44
*TaHsf16*	Trae_2BS_1484A7516	scaffold_146118_2BS: 176,336-179,440	7	3105	2	1	475	5.94	52.08
*TaHsf17*	Trae_2DS_070CE3D50	scaffold_177422_2DS: 92,871-96,205	8	3335	2	1	499	5.7	54.78
*TaHsf18*	Trae_3AL_463ABD4BF	scaffold_196554_3AL: 30,462-32,936	15	2475	2	1	432	5.36	48.37
*TaHsf19*	TRAES3BF029100010CFD	scaffold_223991_3B: 37,660-40,120	15	2461	2	1	441	5.18	49.46
*TaHsf20*	Trae_3DL_8FD0F859B	scaffold_249383_3DL: 45,727-48,075	16	2349	2	1	433	5.35	48.45
*TaHsf21*	Trae_1AL_7D6DC73FC	scaffold_001183_1AL: 39,381-43,295	48	3915	3	2	448	4.91	50.25
*TaHsf22*	nd	scaffold_031159_1BL: 70,097-72,255	9	2159	3	2	445	4.94	49.92
*TaHsf23*	nd	scaffold_061383_1DL: 26,931-29,065	8	2135	3	2	442	5.11	49.70
*TaHsf24*	Trae_6AS_1537629B3	scaffold_487059_6AS: 7273-11,032	7	3760	2	1	458	5.21	49.87
*TaHsf25*	Trae_6BS_25E162197	scaffold_513816_6BS: 34,066-37,539	8	3474	2	1	455	5.33	49.92
*TaHsf26*	Trae_6DS_C59B6322F	scaffold_543918_6DS: 1556-5556	7	4001	2	1	458	5.16	49.86
*TaHsf27*	Trae_1AL_A4B5C1474	scaffold_003124_1AL: 28,946-32,101	30	3156	5	4	368	5	41.70
*TaHsf28*	Trae_1BL_5D8D6B865	scaffold_031443_1BL: 79,599-83,003	27	3405	4	3	364	4.89	41.02
*TaHsf29*	Trae_1DL_B5A84E4C8	scaffold_061579_1DL: 62,790-66,102	29	3313	4	3	370	5.03	42.03
*TaHsf30*	Trae_4AS_52EB860E7	scaffold_307193_4AS: 64,786-67,745	13	2960	2	1	341	5.07	39.63
*TaHsf31*	Trae_4BL_2E125A702	scaffold_321575_4BL: 50,126-53,221	12	3096	2	1	341	5.07	39.59
*TaHsf32*	Trae_4DL_AF19ABC7D	scaffold_342984_4DL: 44,562-50,805	13	6244	4	3	341	5.02	39.49
*TaHsf33*	nd	scaffold_559301_7AL: 9972–11,835	4	1864	5	4	310	4.67	33.78
*TaHsf34*	nd	scaffold_579527_7BL: 16,166-18,111	5	1946	4	3	351	4.94	37.90
*TaHsf35*	nd	scaffold_605087_7DL: 18,736-21,007	5	2272	4	3	351	4.82	37.98
*TaHsf36*	Trae_4AS_02B607421	scaffold_306492_4AS: 132,616-136,430	10	3815	4	3	383	5.22	42.84
*TaHsf37*	Trae_4BL_542B1DA85	scaffold_322416_4BL: 4327-8215	9	3889	4	3	384	5.3	42.87
*TaHsf38*	Trae_4DL_EE941086E	scaffold_344014_4DL: 13,486-17,310	9	3825	4	3	384	5.3	42.92
*TaHsf39*	Trae_5AL_D369204D3	scaffold_374310_5AL: 146,720-151,848	28	5129	2	1	298	9.5	32.14
*TaHsf40*	Trae_5BL_F80E01D65	scaffold_404669_5BL: 141,516-147,139	27	5624	2	1	298	9.31	32.28
*TaHsf41*	Trae_5DL_431CCA490	scaffold_433651_5DL: 31,056-36,542	28	5487	2	1	298	9.2	32.06
*TaHsf42*	Trae_2AL_D3B2C21A7	scaffold_094650_2AL: 33,644-35,170	2	1527	2	1	295	6.12	31.99
*TaHsf43*	nd	scaffold_712376_U: 1–715	1	715	2	1	209	9.5	22.72
*TaHsf44*	nd	scaffold_019033_1AS: 12,760-16,705	25	3946	3	2	404	4.9	42.04
*TaHsf45*	Trae_5BL_FCB1625F3	scaffold_404935_5BL: 109,416-113,225	27	3810	3	2	701	9.22	73.92
*TaHsf46*	nd	scaffold_433530_5DL: 41,946-43,807	26	1862	2	1	397	4.89	41.11
*TaHsf47*	Trae_7AS_937121AF8	scaffold_570040_7AS: 14,527-16,335	6	1809	2	1	374	5.44	40.45
*TaHsf48*	Trae_7BS_03F39ED94	scaffold_592325_7BS: 110,144-112,895	6	2752	3	2	374	5.33	40.33
*TaHsf49*	Trae_7DS_10A9C68FA	scaffold_621446_7DS: 14,666-16,580	6	1915	2	1	367	5.5	39.79
*TaHsf50*	Trae_2DS_01A0E5F7A	scaffold_178567_2DS: 15,585-18,518	4	2934	2	1	320	6.55	35.31
*TaHsf51*	nd	scaffold_642758_U: 53,288-55,875	5	2587	2	1	320	6.66	35.26
*TaHsf52*	nd	scaffold_374067_5AL: 30,626-32,510	7	1885	2	1	388	7.85	41.35
*TaHsf53*	nd	scaffold_404268_5BL: 201,437-203,325	8	1889	2	1	388	7.89	41.46
*TaHsf54*	nd	scaffold_433663_5DL: 11,036-12,820	8	1785	2	1	388	8.42	41.39
*TaHsf55*	nd	scaffold_201352_3AL: 447–1655	0	1209	3	2	277	5.54	31.15
*TaHsf56*	nd	scaffold_194514_3AL: 70,656-72,591	2	1936	2	1	294	6.26	32.57
*TaHsf57*	nd	scaffold_220888_3B: 91,006-92,356	0	1351	2	1	322	5.46	35.55
*TaHsf58*	TRAES3BF021000010CFD	scaffold_220882_3B: 116,126-117,710	2	1585	2	1	325	5.94	35.72
*TaHsf59*	nd	scaffold_249994_3DL: 60,736-62,250	2	1515	2	1	321	6.16	35.38
*TaHsf60*	nd	scaffold_249450_3DL: 110,687-117,555	16	6869	3	2	225	7.11	25.63
*TaHsf61*	nd	scaffold_193607_3AL: 163,384-164,530	13	1147	2	1	236	6.91	26.05
*TaHsf62*	TRAES3BF005500020CFD	scaffold_223354_3B: 26,214-27,330	14	1117	2	1	227	8.35	24.69
*TaHsf63*	nd	scaffold_250779_3DL: 25,456-26,755	11	1300	2	1	241	8.76	26.40
*TaHsf64*	TRAES_3BF025700020CFD_c1	scaffold_231430_3B: 2326-3400	4	1075	1	0	237	5.11	26.12
*TaHsf65*	nd	scaffold_223198_3B: 70,994-72,095	2	1102	2	1	237	5.99	26.61
*TaHsf66*	TRAES3BF025700030CFD	scaffold_224063_3B: 9915-11,221	27	1307	1	0	274	6.98	29.98
*TaHsf67*	Trae_4BL_86572BB6D	scaffold_321958_4BL: 10,751-11,965	9	1215	1	0	264	5.43	29.06
*TaHsf68*	Trae_4BL_F6C3B5069	scaffold_320289_4BL: 21,946-23,120	25	1175	1	0	275	8.4	30.21
*TaHsf69*	Trae_4BL_5091DE58E	scaffold_320289_4BL: 33,386-34,490	2	1105	1	0	257	4.88	28.50
*TaHsf70*	nd	scaffold_320675_4BL: 112,161-113,540	5	1380	2	1	273	5.7	29.45
*TaHsf71*	nd	scaffold_344468_4DL: 19,506-20,710	14	1205	1	0	276	6.46	30.27
*TaHsf72*	Trae_4DL_FA07D8414	scaffold_343739_4DL: 22,666-23,885	4	1220	1	0	276	5.32	29.85
*TaHsf73*	nd	scaffold_376864_5AL: 4896-6010	4	1115	1	0	273	6.15	30.17
*TaHsf74*	nd	scaffold_375679_5AL: 69,576-70,900	15	1325	1	0	229	5.08	25.56
*TaHsf75*	nd	scaffold_641118_U: 187,271-188,375	0	1105	2	1	268	5.69	29.99
*TaHsf76*	Trae_7AL_6931AA68B	scaffold_558532_7AL: 22,876-24,495	13	1620	2	1	266	6.44	28.23
*TaHsf77*	nd	scaffold_577398_7BL: 12,506-14,356	14	1851	2	1	244	5.61	26.12
*TaHsf78*	nd	scaffold_609477_7DL: 1–1636	16	1636	2	1	263	6.11	28.04

### Conserved domains analysis of *TaHsf*

We identified five conserved domains by sequence alignment approaches (Table 2). All the TaHsf predicted proteins contained a highly conserved DBD domain, forming with a three helical bundles (H1, H2 and H3) and four-stranded antiparallel β-sheet in their N-terminal regions. However, within the *Hsf* family, the length of the DBD domain was quite different. We then used the MARCOIL tool to detect the presence of a property of the HR-A/B, the coiled-coil structure characteristic of leucine zipper-type protein interaction domains. We found that most of the TaHsfs proteins consisted of NES and NLS domains, which are vital for shuttling *Hsfs* between the nucleus and cytoplasm. As was expected in the A-type *TaHsfs*, additional sequence comparisons identified AHA domain in the middle of the C-terminal activation domains. By contrast, these domains were not detected in the B- and C-type *TaHsfs*. To further predict and verify domains in the *TaHsf*s proteins, we used the Multiple EM for Motif Elicitation (MEME) motif search tool. Using this, we found thirty corresponding consensus motifs (Additional file 1: Figure S1, Additional file 2). Compared with class B and C, the members of class A contained the greatest number of conserved motifs (22), with the majority (12) detected in *TaHsf1* and *TaHsf3*. The conserved motifs 1, 2, 4, 5, 8 16 represented the DBD domain. Motif 1 was found in 77 members of TaHsf family (except for *TaHsf33*). Regarding coiled-coiled structures, motif 3 was detected in class A and class C *TaHsfs* family, while motif 7 was detected in class B. The conserved motifs 10, 20, 22, 23, 25, 28, 30 were identified as NLS domains. Motifs 10 and 25 represented NLS domains in class A, whereas NLS domains were represented by motifs 20, 23, 28 and 30 in class B, motifs 22 and 23 in class C. Motif 15 represented NES domains, and motif11 was identified as characteristic AHA domains. Thus through the combination of the two methods, predicted DBD domains and HR-A/B domains were observed in each *TaHsfs* and varied greatly in size and sequence.

**Table 2:** Functional domains of TaHsfs

Names	Protein type (A-B-C)	DBD	HR-A/B	NLS	NES	AHA
*TaHsf1*	A1a	38–128	163–227	(245)RRIVAANKKRR	(508)LTEQMGLL	AHA2(464)DSFWEQFLCA
*TaHsf2*	A1a	1–73	109–173	(191)RRIVAANKKRR	(458)LTEQMGLL	AHA2(414)DSFWEQFLCA
*TaHsf3*	A1a	1–91	125–189	(207)RRIVAANKKRR	(474)LTEQMGLL	AHA2(430)DSFWEQFLCA
*TaHsf4*	A2a	38–128	143–207	(223)RKELEDAISNKRRRR	nd	AHA1(313)DDFWEDLL
*TaHsf5*	A2a	40–130	145–209	(225)RKELEDAISNKRRRR	nd	AHA1(315)DDFWEDLL
*TaHsf6*	A2a	45–135	150–214	(230)RKELEDAISNKRRRR	nd	AHA(320)DDFWEDLL
*TaHsf7*	A2b	43–133	149–213	(229)RKELHDAISKKRRRR	(400)KMGYL	AHA1(370)DNFWEELL
*TaHsf8*	A2b	44–134	150–214	(230)SKELHDAISKKRRRR	(392)KMGYF	AHA1(362)DNFWEGLL
*TaHsf9*	A2b	43–133	149–213	(229)RKELHDAISKKRRRR	(399)KMGYL	AHA1(369)DNFWEELL
*TaHsf10*	A2b	42–132	148–212	(228)RKELHDAMSKKRRRS	nd	nd
*TaHsf11*	A2b	41–131	147–211	(227)RKELHDAMSKKRRRS	nd	AHA1(353)DDFWEELM
*TaHsf12*	A2e	66–156	178–242	(260)RKELAEALLSKKRGR	nd	AHA1(314)ESFWKELL
*TaHsf13*	A2e	66–156	180–244	(262)RKELAEALLSKKRGR	nd	AHA1(320)ESFWKELL
*TaHsf14*	A3	49–139	175–221	(248)RVKRKFLKHV	nd	nd
*TaHsf15*	A3	84–174	210–256	(283)RVKRKFLKHV	nd	nd
*TaHsf16*	A3	80–170	206–252	(279)RVKRKFLKHV	nd	nd
*TaHsf17*	A3	81–171	207–253	(280)RVKRKFLKHV	nd	nd
*TaHsf18*	A4a	13–103	126–183	(198)KKRR	(419)MTEKLGHL	AHA1(244)LNSLENFFKE AHA2(370)DGFWQQFLTE
*TaHsf19*	A4a	13–103	126–183	(198)KKRR	(428)MTEKLGHL	AHA1(244)LNSLENFFKE AHA2(379)DGFWQQFLTE
*TaHsf20*	A4a	13–103	126–183	(198)KKRR	(420)MTKKLGHL	AHA1(244)LNSLENFFKE AHA2(370)DGFWQQFLTE
*TaHsf21*	A4d	25–115	138–195	(220)KKRR	(430)ITQQMGHL	AHA1(267)LVSMEKLVQR AHA2(386)DLFWERFLTD
*TaHsf22*	A4d	23–113	136–193	(219)KKRR	(432)ITEQMGHL	AHA1(267)LVSMEKLVRR AHA2(388)DLFWERFLTD
*TaHsf23*	A4d	23–113	136–193	(218)KKRR	(429)ITEQMGHL	AHA1(270)LVSMEKLVQR AHA2(385)DLFWERFLTD
*TaHsf24*	A5	20–111	131–188	(199)KMAEASSMFADALHKK	nd	(414)DNFWEQFLTE
*TaHsf25*	A5	20–111	131–188	(199)KMAEASSMFADALHKK	nd	(414)DNFWEQFLTE
*TaHsf26*	A5	20–111	131–188	(199)KMAEASSMFADALHKK	nd	(414)DNFWEQFLTE
*TaHsf27*	A6a	52–142	159–223	(238)KRKELEDAISKKRRR	(352)IDELGQQLGYL	(322)SDFWAELFSD
*TaHsf28*	A6a	48–138	155–219	(234)KRKELEDAISKKRRR	(348)IDELAQQLGYL	(318)NDFWAELFSD
*TaHsf29*	A6a	54–144	161–225	(240)KRKELEDAISKKRRR	(354)IDELAQQLGYL	(324)NDFWAELFSD
*TaHsf30*	A6b	46–136	153–217	(232)KLKDLEDGYPTKRRR	nd	(311)DDFWEELLSE
*TaHsf31*	A6b	46–136	153–217	(232)KLKDLEDGYPTKRRR	nd	(311)DDFWEELLSE
*TaHsf32*	A6b	46–136	153–217	(232)KLKDLEDAYSNKRRR	nd	(311)DDFWEELLSE
*TaHsf33*	A7b	47–138	150–175	nd	nd	(246)TDMIWYELL
*TaHsf34*	A7b	49–139	173–223	nd	nd	(298)TDMIWYELL
*TaHsf35*	A7b	49–139	173–223	nd	nd	(298)TDMIWYELL
*TaHsf36*	A8	37–127	173–230	nd	nd	nd
*TaHsf37*	A8	37–127	173–230	nd	nd	nd
*TaHsf38*	A8	37–127	173–230	nd	nd	nd
*TaHsf39*	B1	27–117	172–209	nd	nd	nd
*TaHsf40*	B1	30–120	174–211	nd	nd	nd
*TaHsf41*	B1	30–120	174–211	nd	nd	nd
*TaHsf42*	B2a	13–103	157–193	(223)KRSRE	nd	nd
*TaHsf43*	B2a	26–116	170–206	nd	nd	nd
*TaHsf44*	B2c	42–132	215–251	(321)KRARD	nd	nd
*TaHsf45*	B2c	176–266	349–385	(455)KRARD	nd	nd
*TaHsf46*	B2c	42–132	215–251	(321)KRARD	nd	nd
*TaHsf47*	B2d	32–122	192–228	(300)KRMRH	nd	nd
*TaHsf48*	B2d	32–122	192–228	(300)KRMRH	nd	nd
*TaHsf49*	B2d	32–122	192–228	(293)KRMRH	nd	nd
*TaHsf50*	B4b	40–130	201–237	(299)KKKR	nd	nd
*TaHsf51*	B4b	39–129	200–236	(299)KKKR	nd	nd
*TaHsf52*	B4c	26–117	207–243	(336)PVGA	(362)LALENDDL	nd
*TaHsf53*	B4c	26–117	207–243	(336)PVGA	(362)LALESDDL	nd
*TaHsf54*	B4c	26–117	207–243	(336)PVGA	(362)LALESDDL	nd
*TaHsf55*	C1a	21–111	121–164	nd	nd	nd
*TaHsf56*	C1a	1–84	121–171	nd	nd	nd
*TaHsf57*	C1a	21–111	154–197	nd	nd	nd
*TaHsf58*	C1a	25–115	159–202	nd	nd	nd
*TaHsf59*	C1a	25–115	159–202	nd	nd	nd
*TaHsf60*	C1a	21–111	149–192	nd	nd	nd
*TaHsf61*	C1b	19–109	126–169	nd	nd	nd
*TaHsf62*	C1b	19–109	131–174	nd	nd	nd
*TaHsf63*	C1b	19–109	131–174	nd	nd	nd
*TaHsf64*	C2a	1–75	97–140	(168)KRPR		
*TaHsf65*	C2a	24–114	134–177	(202)KRPR	nd	nd
*TaHsf66*	C2a	19–109	132–175	(203)KRPR	nd	nd
*TaHsf67*	C2a	12–102	124–167	(195)QRPR	nd	nd
*TaHsf68*	C2a	21–111	135–178	(206)KRPR	nd	nd
*TaHsf69*	C2a	20–110	132–175	(203)KKPR	nd	nd
*TaHsf70*	C2a	24–114	135–178	(205)KRPR	nd	nd
*TaHsf71*	C2a	23–113	135–178	(206)KRRR	nd	nd
*TaHsf72*	C2a	24–114	141–184	(211)KRPR	nd	nd
*TaHsf73*	C2a	30–120	143–186	(207)NRPR	nd	nd
*TaHsf74*	C2a	1–84	106–149	(177)KRPR	nd	nd
*TaHsf75*	C2a	23–113	132–175	(198)KRLR	nd	nd
*TaHsf76*	C2b	15–105	132–175	(204)KRAR	nd	nd
*TaHsf77*	C2b	1–84	97–153	nd	nd	nd
*TaHsf78*	C2b	13–103	129–172	(201)KRAR	nd	nd

*DBD* DND-binding domain, *HR-A/B* OD (oligomerisation domain), heptad pattern of hydrophobic amino acid residues; NLS: Nuclear localization signal, *NES* Nuclear export signal. *AHA* Activator motifs, **a**romatic (W, F, Y), larger **h**ydrophobic (L, I, V) and **a**cidic (E, D) amino acid residues; Numbers in brackets reveals the position of the first amino acid present in the putative NLS, NES, and AHA in the C-terminal; nd: no domains detectable by sequence similarity

### Phylogenetic analysis in wheat *Hsf* proteins

**Fig. 1:**
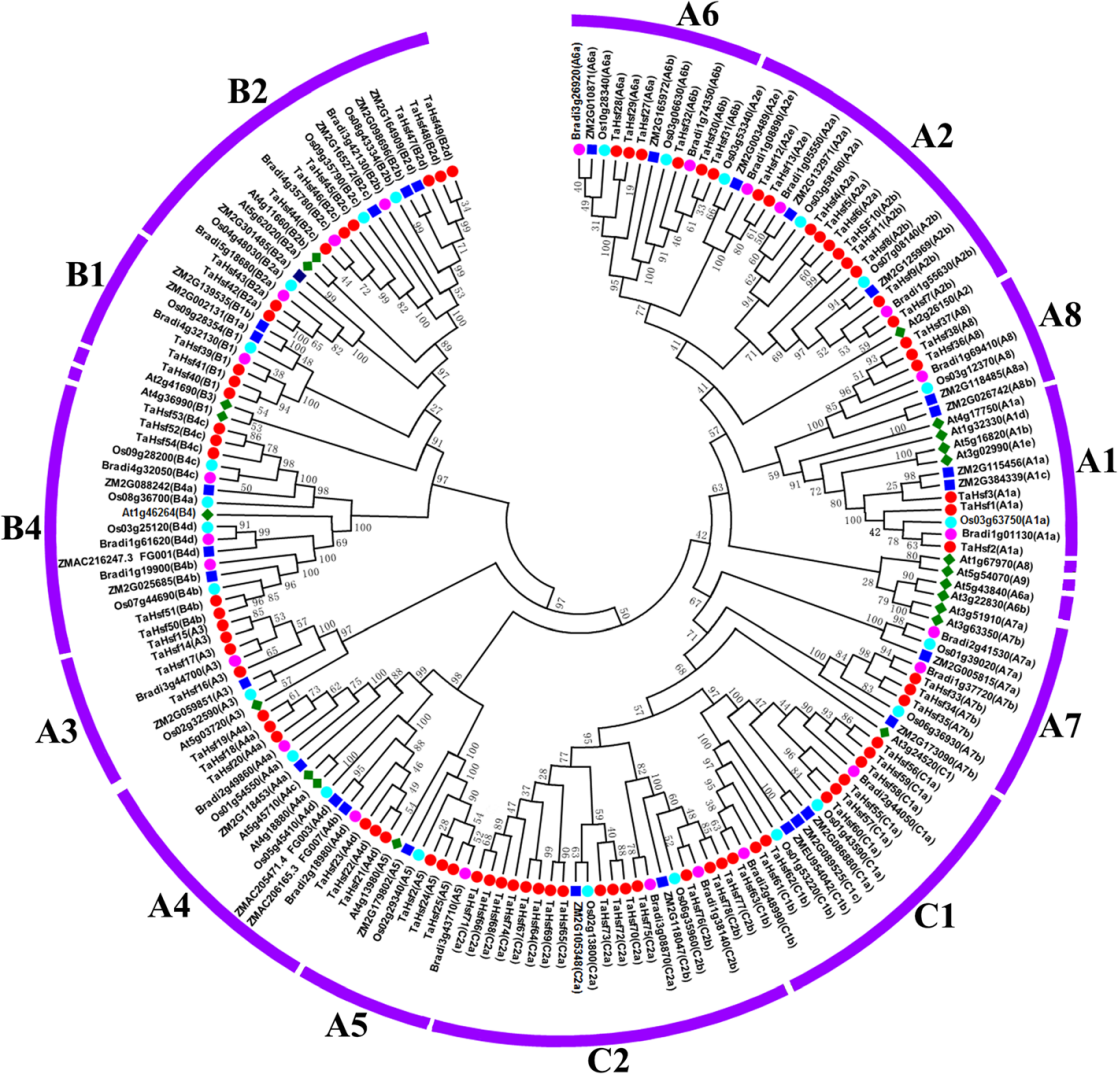
Phylogenetic tree of Hsf proteins from wheat, Arabidopsis, rice, brachypodium and maize. The N-proximal regions (from the start of the DNA-binding domain to the end of the HR-A/B region) of *Hsf* proteins were used to construct an unrooted neighbor-joining tree with MEGA6.0 (with pairwise deletion and Poisson correct). For *Hsf* proteins of *Arabidopsis* (prefixed by AT), *rice* (prefixed by Os), *Brachyposium* (prefixed by Bradi) and *maize* (prefixed by ZM), both locus ID and subclass numbers are given. *TaHsf* proteins are marked in red

To further evaluate the phylogenetic relationships amidst *Hsf* families, the *Hsf* conserved amino acid sequences (from the beginning of the DNA-binding domain to the end of the HR-A/B region) of 39 proteins from wheat (*Triticum aestivum L.*), 21 proteins from Arabidopsis (*A. thaliana*), 25 from rice (*O. sativa*), 24 from brachypodium (*B. distachyon*) and 30 from maize (*Z. mays*) were used to construct a phylogenetic tree (Fig. 1). According to this tree, class *HsfA* showed the maximum number of subclasses among the three major groups, and contained eight smaller clusters of which five (A6, A2, A8, A1 and A7) were closer to class *HsfC* than class HsfA3, A4 and A5. Two *HsfA6* members from *Arabidopsis* (At5g43840 and At3g22830) were not clustered with the *HsfA6* subclass from other plant species, but were closer to the *HsfA7* subclass. *Brachypodium* Hsfs were closer to wheat *Hsf* proteins compared with *Arabidopsis*, *maize* and *rice*, which was in line with the botanical classification.

### Genome distribution and gene duplication of *TaHsf* gene family

**Fig. 2:**
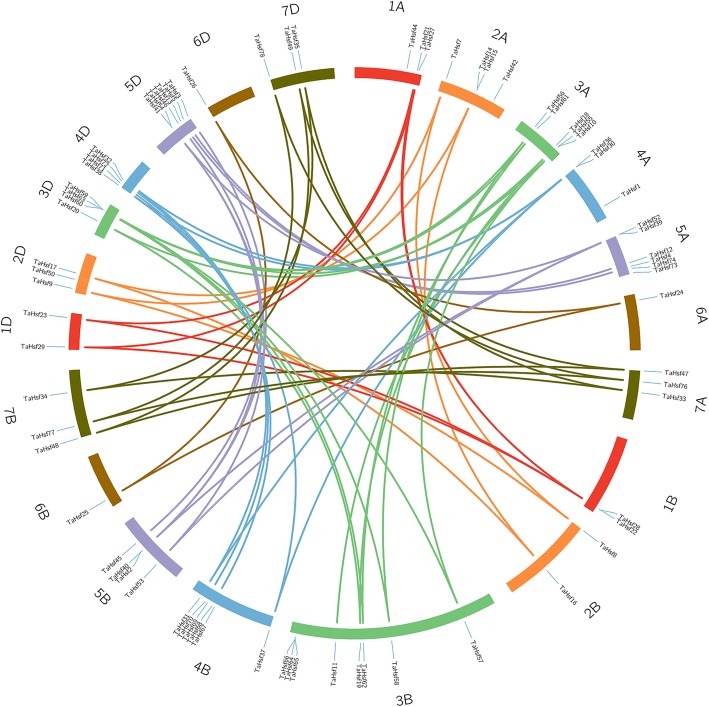
Chromosomal localizations and the homologous *TaHsf* genes in wheat A, B and D sub-genomes

We next determined chromosomal locations of *TaHsf* genes by leveraging the available wheat genome annotation information (Fig. 2). A total of 25, 26 and 23 *TaHsf* genes are found in the A, B and D sub-genomes, respectively (B > A > D). The distribution of *Hsf* genes was not even across the chromosomes. There were 7, 9, 17, 13, 16, 3 and 9 genes in the group 1 to group 7 chromosomes, which reveal obvious differences between group 3, 4, 5 and other four groups. Chromosome 3B had the highest number of *Hsf* genes with 8, while chromosome 6A, 6B and 6D all had only one *Hsf* gene eachs. These results suggest that *Hsf* gene duplication events may have happened in wheat 3, 4 and 5 group chromosomes during wheat formation and the evolution of gene families in the different sub-genome is independent, which may relate to gene function.

**Fig. 3:**
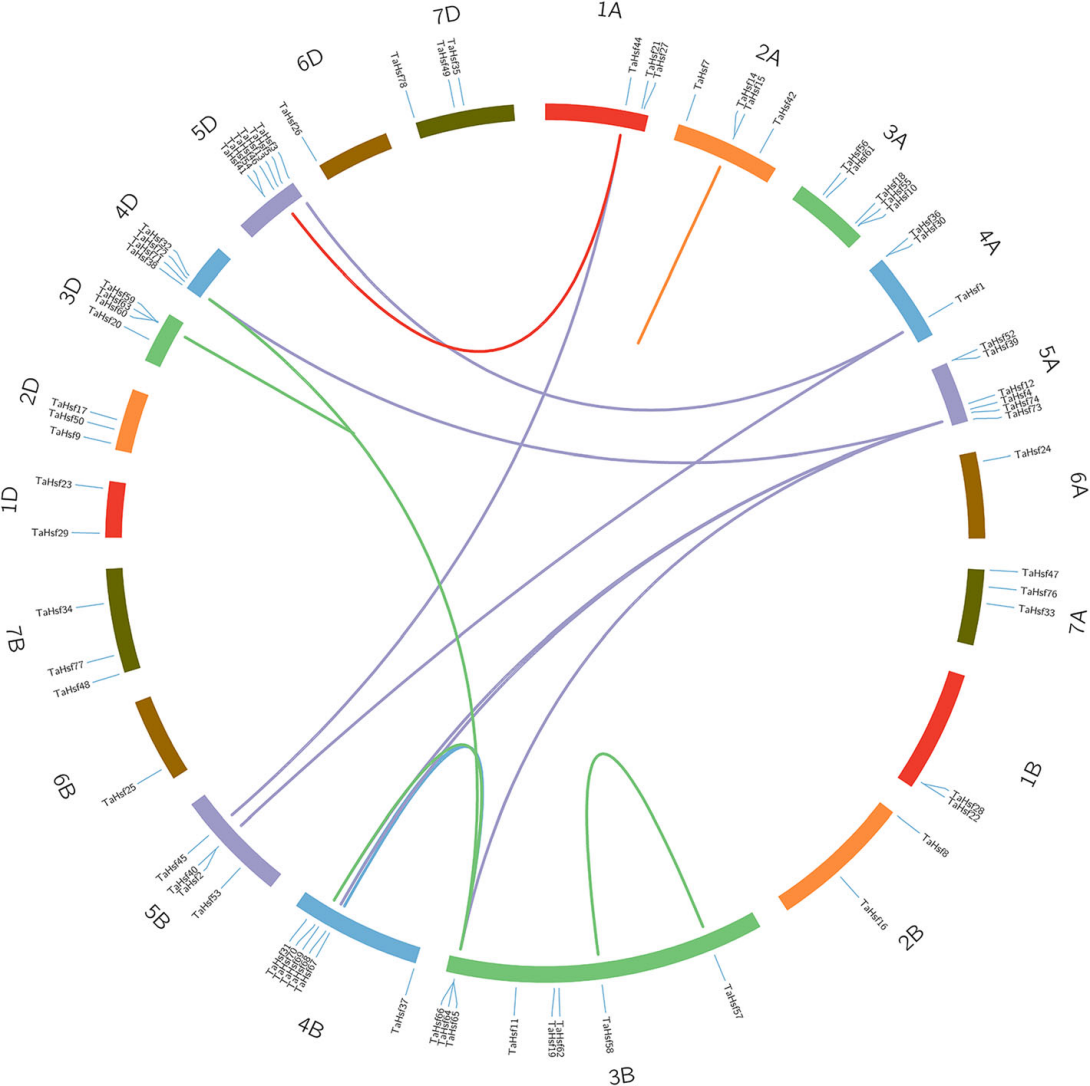
Duplicated *Hsf* gene pairs identified in *wheat*. Seven homologous groups of wheat chromosomes are depicted in different colors. Duplicated gene pairs are depicted in corresponding colors and linked using lines with the corresponding color

Gene duplication is frequently revealed in plant genomes, resulting from polyploidization or through tandem and segmental duplication related to replication [30]. Here, we found 17 homologous gene groups with a copy on each of A, B and D homologous chromosome, and 7 gene pairs with a copy on only 2 of the 3 homologous chromosomes, while the other 13 genes were not found as homologs (Fig. 2, Additional file 3). Our results indicate that gene loss may happen throughout the wheat *Hsf* gene family, leading to the loss of some homologous copies. Moreover, these homologous genes are clustered in group 3, 4 and 5 chromosomes, which was in line with the above analysis of chromosome localization, suggesting that group 3, 4 and 5 chromosomes subjected less sequence loss and interaction impact compared to other homologous chromosome groups. In addition, 17 pairs of duplicated genes from different sub-genomes were also found, containing 3 duplication events in the same chromosome and 14 segmental duplication events between different chromosomes, indicating that the duplication events could play important roles in the extension of the *Hsf* genes in wheat genome (Fig. 3, Additional file 3).

### Phylogenetic analysis of *Hsfs* between the *T. urartu, A. tauschii,* and wheat orthologs

**Fig. 4:**
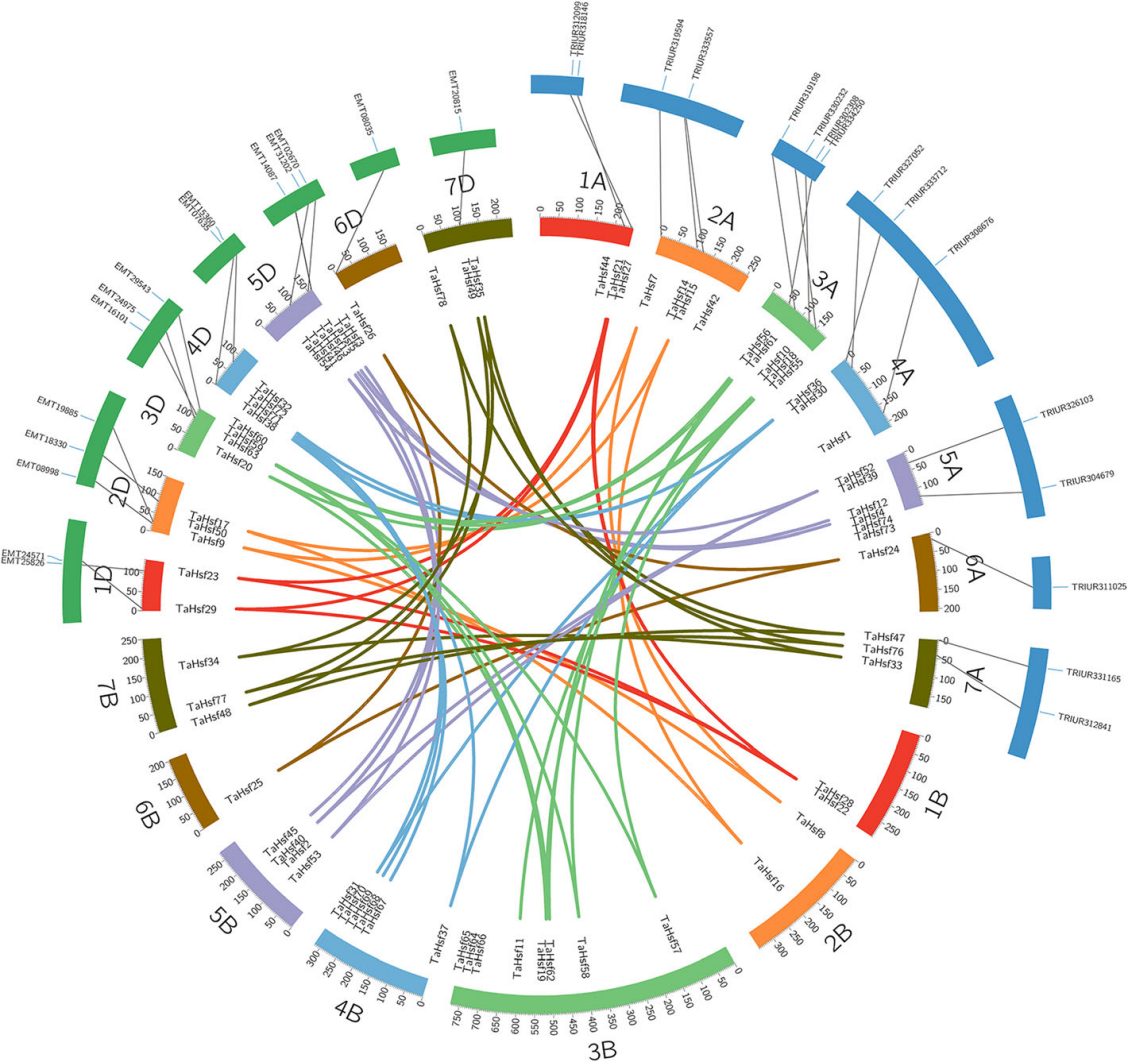
Collinear analysis for the *Hsf* gene family among *wheat*, *T.urartu* and *A.tauschii.* The green annulus on the top left represent chromosomes of *A. tauschii* and the blue annulus on the top right represent chromosomes of *T. urartu*. Different colors represent seven homologous groups of wheat chromosomes. Homeologous genes of each group are linked by lines with corresponding color

We also identify the *Hsfs* gene in the diploid ancestors of wheat, *T. urartu* and *A. tauschii*, to investigate the change of *Hsf* number in transition from diploidy to hexaploidy within a given subgenome. Results showed that 16 and 15 putative *Hsfs* were identified in *T. urartu* and *A. tauschii* through our methods, respectively (Additional file 4). Total 16 *T. urartu*-*Hsfs,* 25 *T. aestivum*-A-*Hsfs,* 15 *A. tauschii*-*Hsfs, and* 23 *T. aestivum*-D-*Hsfs* gene sequences were applied to build gene trees. 16 pairs of *T. urartu*-wheat A genome orthologs were mapped to *T. urartu* chromosomes with 2 on 1A, 2 on 2A, 4 on 3A, 3 on 4A, 2 on 5A, 1 on 6A and 2 on 7A (Fig. 4). 15 pairs of *A. tauschii*-wheat D genome orthologs were mapped to *A.tauschii* chromosomes with 2 on 1D, 3 on 2D, 3 on 3D, 2 on 4D, 3 on 5D, 1 on 6D and 1 on 7D (Fig. 4). The majority of the orthologs (75 and 66.67% for *T. urartu* and *A. tauschii*, respectively) belonged to class A, as expected due to the high proportional composition of this type (48.72%) among the identified wheat *Hsf* genes. Moreover, the chromosome locations of the majority of wheat *Hsf* genes and their orthologs in *T. urartu* and *A. tauschii* corresponded to one another (Additional file 5).

### Modulatory network between *TaHsf* genes with other wheat genes

**Fig. 5:**
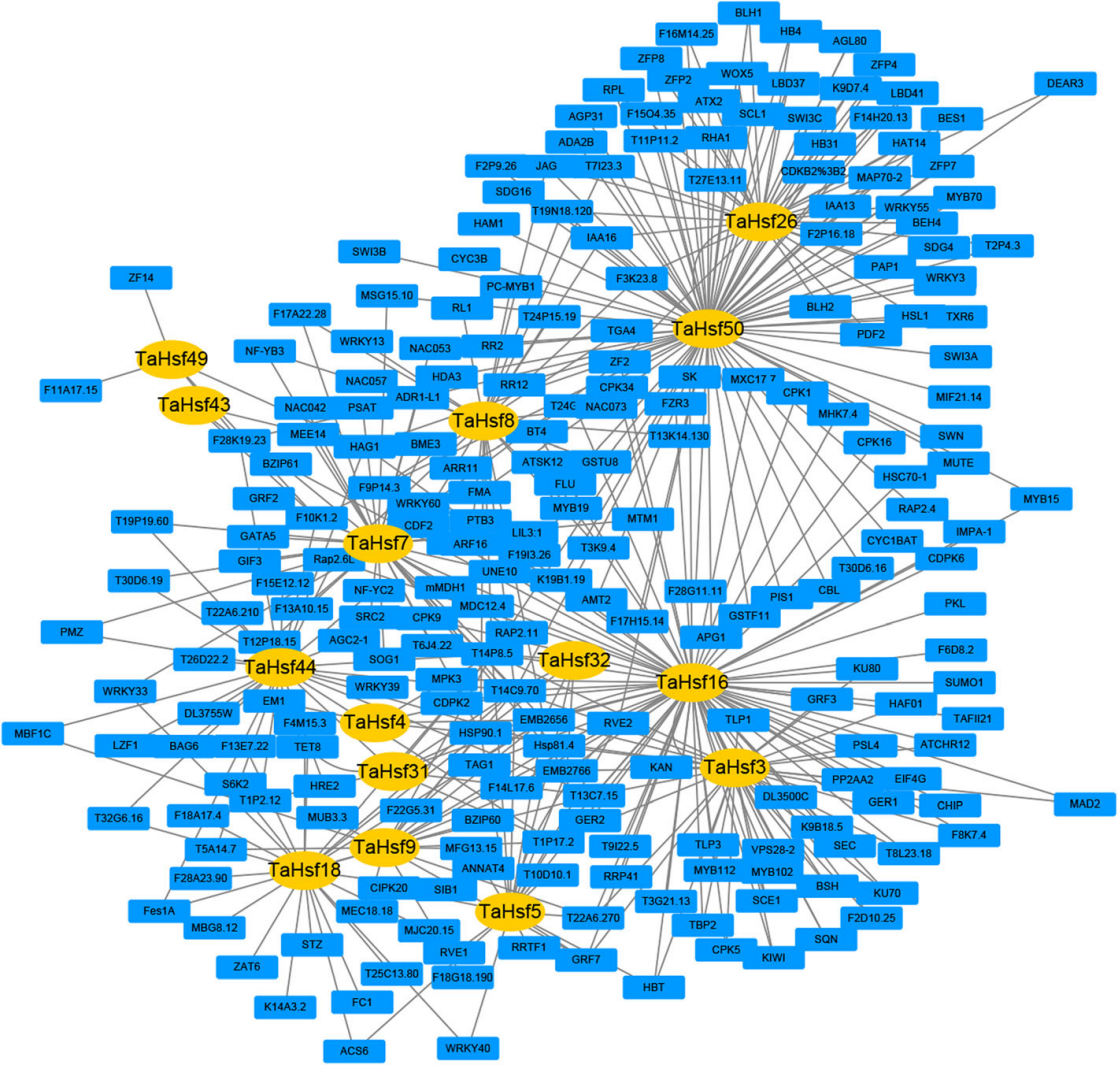
An interaction network of *TaHsf* genes in *wheat* based on the orthologs in *Arabidopsis*. Fifteen *TaHsfs* were found to have homology with Arabidopsis genes and the 420 gene pairs of network interactions

**Fig. 6:**
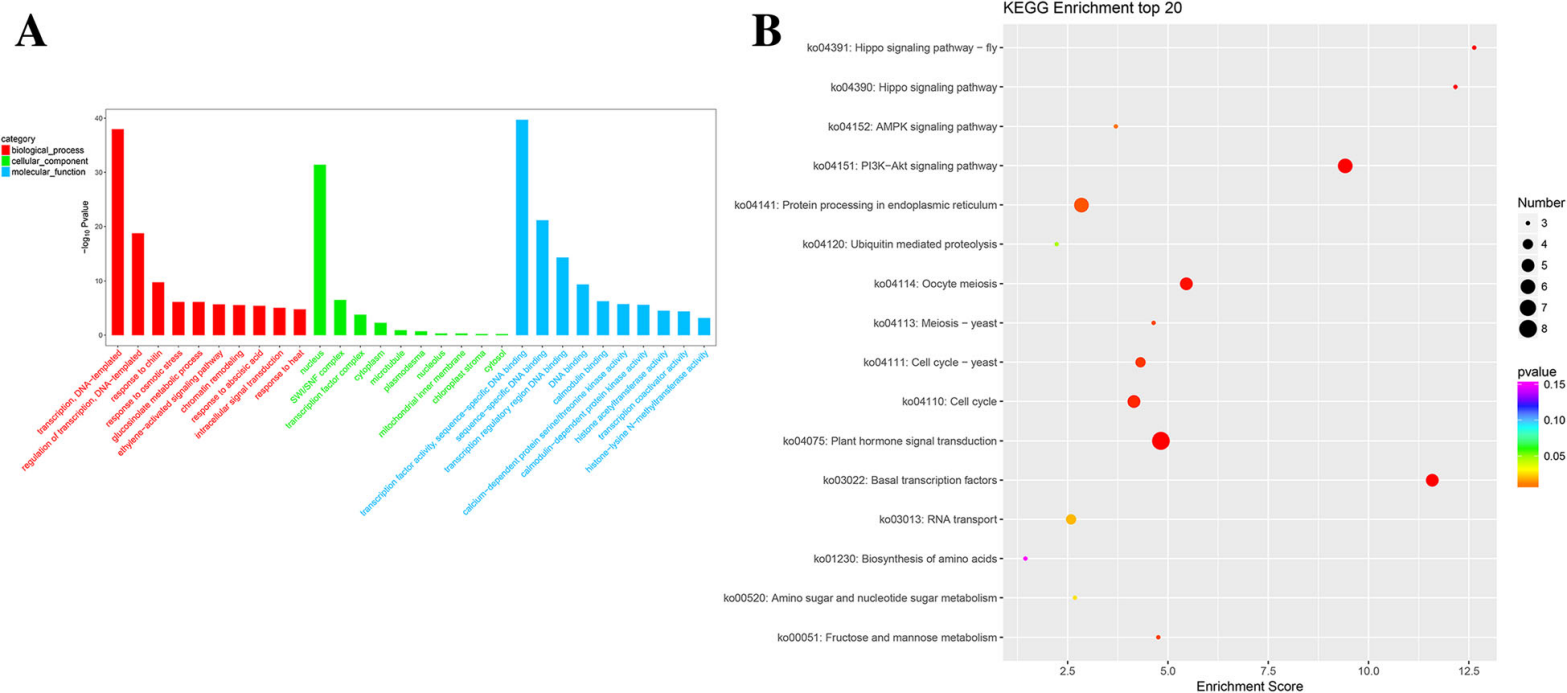
Functional and KEGG pathway categories of 15 *TaHsfs* interacting with *wheat* genes. **a** Top 10 GO categories that are enriched in 15 *TaHsfs* interacting with wheat genes according to –log_10_Pvalues. GOs included biological process, cellular component and molecular function. **b** Top 20 KEGG pathways that are enriched in 15 *TaHsfs* interacting with *wheat* genes according to enrichment scores

In order to comprehend the interactions between *TaHsfs* and other wheat genes, the modulatory network of them (Fig. 5) was predicted via the orthology-based method [31]. Results showed that 15 *TaHsfs* were shown to have homology with *Arabidopsis* genes and the 420 gene pairs of network interactions were found with the average of 28 gene per *TaHsf*, suggesting the *TaHsfs* were broadly engaged in the regulatory network and biological process in wheat. Among these, 292 genes interacted with T*aHsfA* and 128 genes interacted with *TaHsfB*. *TaHsf16* (*A3*) was found to interact with 77 wheat genes, including *Hsp81.4*, *ZF2*, *HBT* and *HSP90.1*, suggesting it was mainly participated in response to stress, metal ion binding, cell differentiation and protein folding. *TaHsf18* (*A4a*) was found to interact with 24 wheat genes, including *ZAT6*, *STZ* and *S6K2*, suggesting it was mainly engaged in metal ion binding, intracellular signal transduction and negative regulation of cell proliferation. *TaHsf50* (*B4b*) was predicted to interact with 88 wheat genes, including *MYB15*, *MYB70*, *ZFP2*, *FMA*, and *HB31*, suggesting it is engaged primarily in the regulation of transcription, asmonic acid, metal ion binding and DNA binding. *TaHsf44* (*B2c*) was found to interact with 30 wheat genes including *AGC2–1*, *WRKY39*, *BAG6* and *NF-YC2*, suggesting it is mainly engaged in defense response, calmodulin binding, response to heat and flower development (Additional files 6, 7). Moreover, GO and KEGG pathway descriptions of those interacting genes were analyzed to understand the potential function and pathway of the 15 *TaHsfs* (Fig. 6). The 15 *TaHsf* interacting genes were significantly enriched for transcription, DNA-templating, response to heat, transcription factor activity, sequence-specific DNA binding and calmodulin binding (Fig. 6a). Significantly enriched pathways included plant hormone signal transduction, *PI3K-Akt* signaling pathway, and protein processing in endoplasmic reticulum (Fig. 6b).

### Tissue-specific expression patterns of *TaHsf* genes

**Fig. 7:**
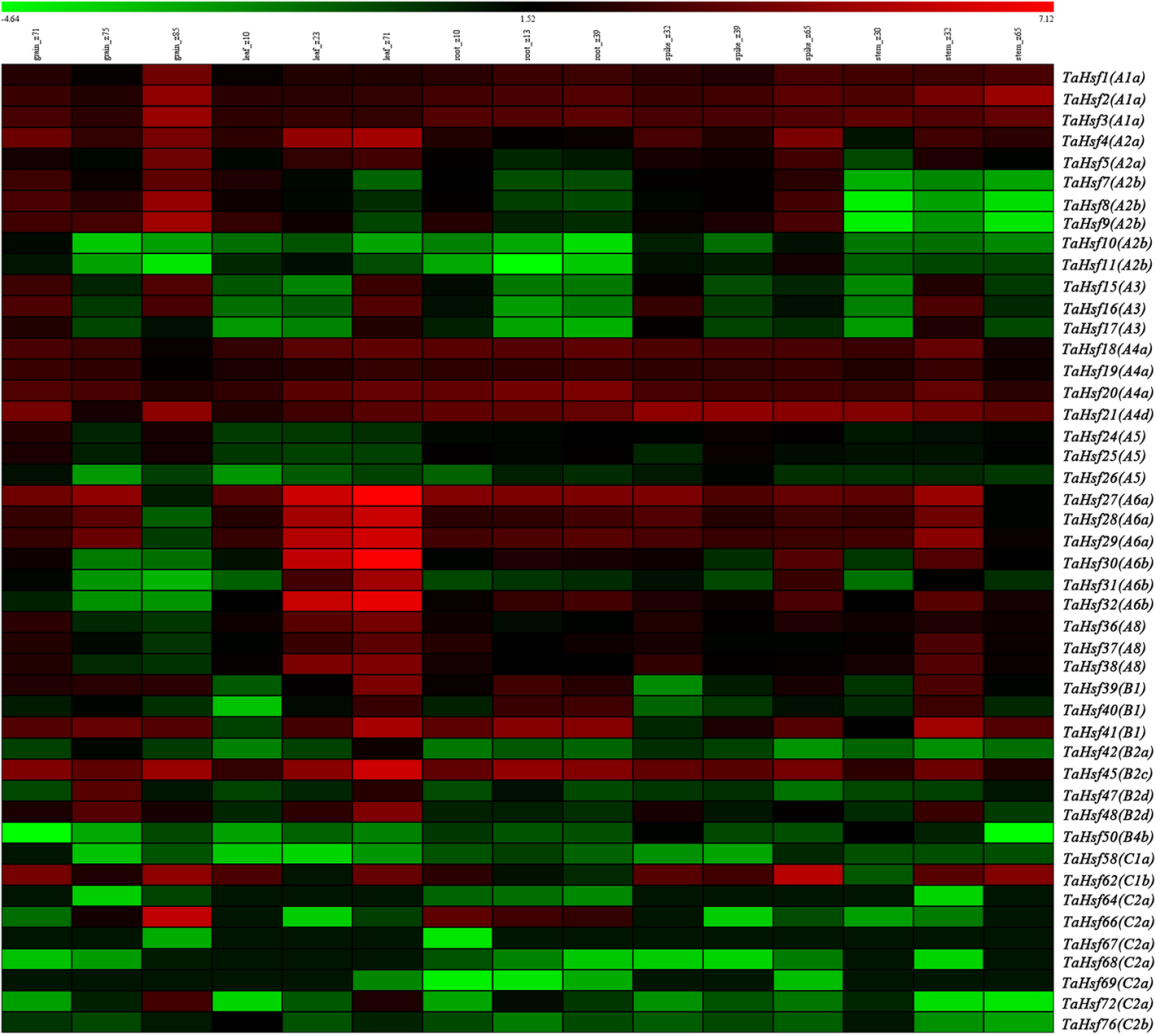
Heat map of the expression profiles of 46 *TaHsf* genes in five different tissues (grain, leaf, root, spike and stem). Log2 transformed FPKM values were used to create the heat map. The red or green colors stand for the higher or lower relative abundance of each transcript in each sample. Z represent Zadoks scale, a decimal code for the growth stages of cereals. *P*-value< 0.05 were regarded as statistically significant

Using available RNA-seq data for five different tissues, the tissue specificity of the *TaHsf* genes was investigated to focus on the temporal and spatial expression patterns and putative functions of *Hsf* genes in wheat growth and development. According to FPKM values, we found that the expression levels of the *TaHsfs* varied significantly in different tissues (Fig. 7). *TaHsf10 (A2b)*, *TaHsf15 (A3)*, *TaHsf16 (A3)*, *TaHsf17 (A3)*, *TaHsf30 (A6b)*, *TaHsf32 (A6b)*, *TaHsf50 (B4b)*, *TaHsf58 (C1a)*, *TaHsf66 (C2a)* and *TaHsf72 (C2a)* exhibit low expression abundance in endosperm, inner pericarp and outer pericarp, while TaHsf1 (A1a), *TaHsf2 (A1a)*, *TaHsf3 (A1a)*, *TaHsf4 (A2a)*, *TaHsf8 (A2b)*, *TaHsf9 (A2b)*, *TaHsf20 (A4a)*, *TaHsf21 (A4d)*, *TaHsf36 (A8)* and *TaHsf41 (B1)* had high expression abundances. Furthermore, the expression levels of the TaHsfs varied significantly in different grain layers over development (Additional file 1: Figure S2).

### Expression patterns of *TaHsf* genes under abiotic stresses

**Fig. 8:**
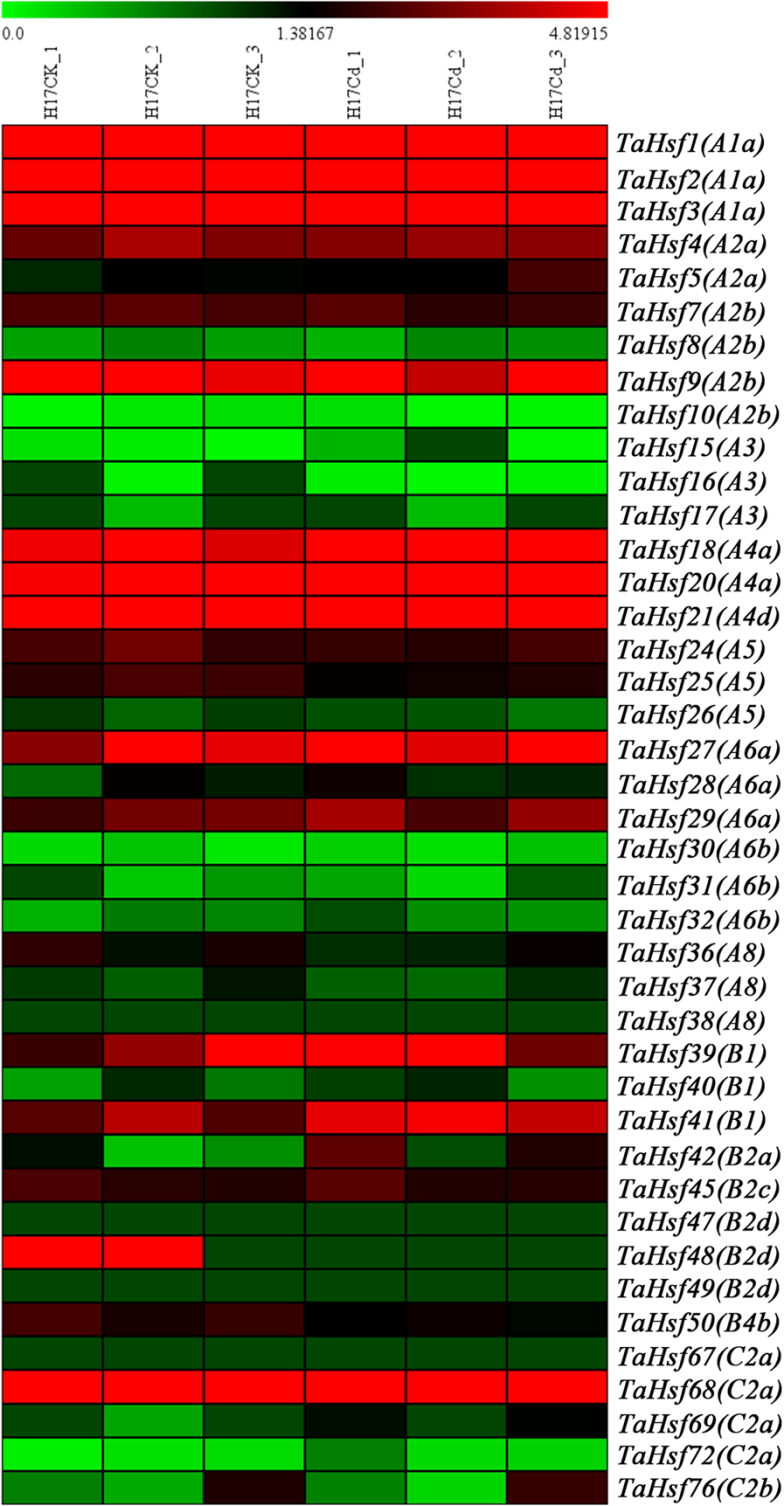
Heat map of the expression profiles of *TaHsf* genes under Cd treatment. FPKM values were used to create the heat map. The red or green colors indicate the higher or lower relative abundance or each transcript in each sample

To study the roles of *TaHsf* genes in response to abiotic stresses, expression of all *TaHsf* genes in response to drought, heat, and Cd stress was investigated using RNA sequencing data. All 46 wheat *Hsf* genes revealed different expression patterns under these dynamic conditions. Among them, the expression levels of *TaHsf2 (A1a)* and *TaHsf21 (A4d)* were both down-regulated under drought, heat, drought and heat stresses, while the expression of *TaHsf4 (A2a)*, *TaHsf15 (A3)*, *TaHsf16 (A3)*, *TaHsf17 (A3)*, *TaHsf28 (A6a)* and *TaHsf41 (B1)* was up-regulated (Additional file 1: Figure S3). According to our RNA sequencing data (Additional file 8) [31], expression levels of *TaHsf3 (A1a)*, *TaHsf4 (A2a)*, *TaHsf5 (A2a)*, *TaHsf16 (A3)*, *TaHsf18 (A4a)*, *TaHsf20 (A4a)*, *TaHsf31 (A6b)* and *TaHsf32 (A6b)* were up-regulated under Cd stress, while the expression of *TaHsf7 (A2b)*, *TaHsf8 (A2b)*, *TaHsf9 (A2b)*, *TaHsf26 (A5)* and *TaHsf50 (B4b)* was down-regulated (Fig. 8).

### Verification of the expression of *TaHsf* in wheat under cd stress by qRT-PCR

**Fig. 9:**
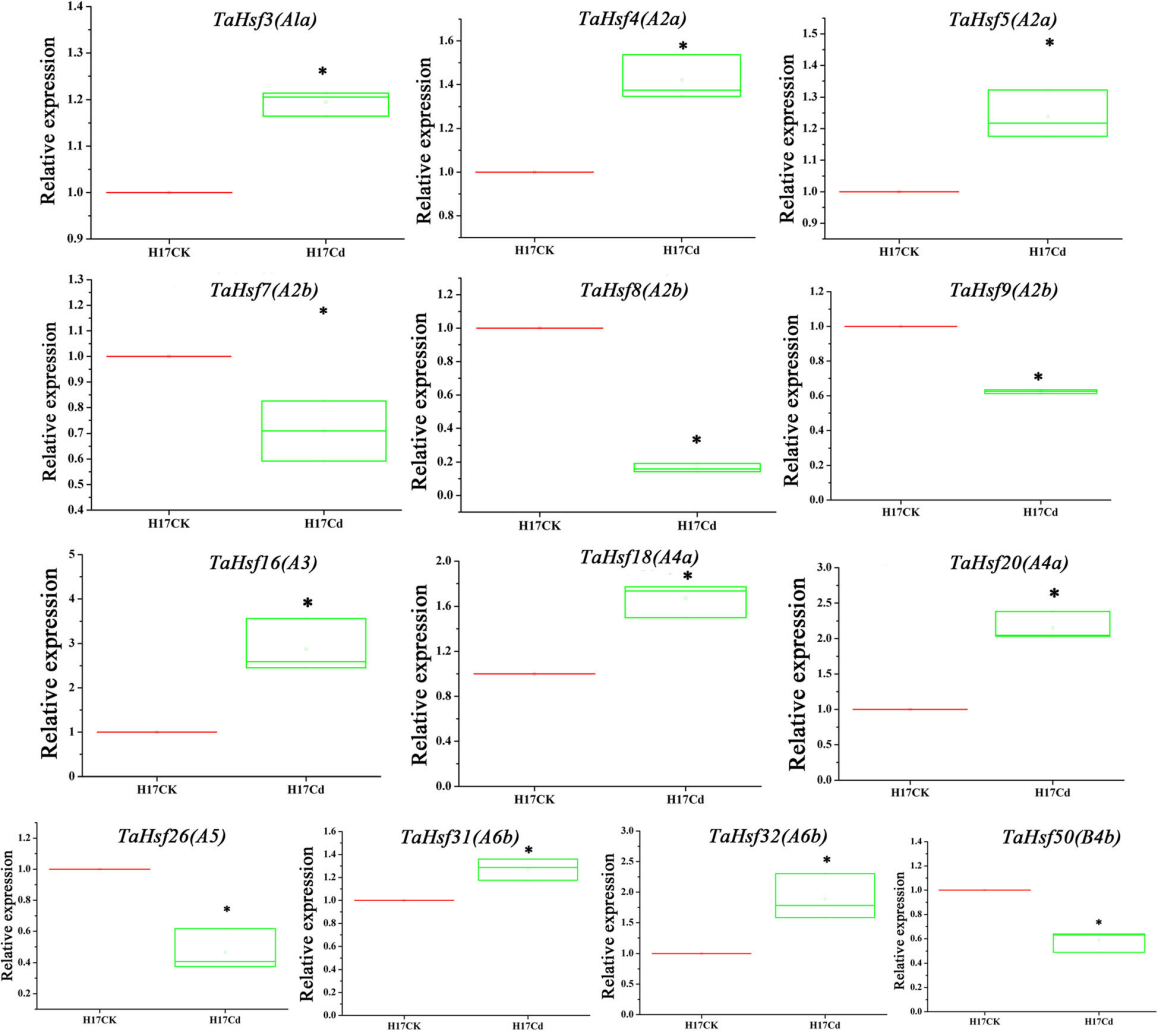
Verification of the expression level of *TaHsfs* by qRT-PCR analysis. Relative expression levels of 13 *TaHsfs* under Cd treatment. * represents *P* < 0.05 vs H17CK

According to the expression analysis based on diverse RNA sequencing data above, we obtained an overview of expressed *TaHsfs* under various agriculturally-relevant stressors. To further verify these results we selected a subset of these *TaHsfs* to detect their expression levels in root under Cd stress through qRT-PCR. Results showed that compared with H17CK group, levels of *TaHsf3 (A1a)*, *TaHsf4 (A2a)*, *TaHsf5 (A2a)*, *TaHsf16 (A3)*, *TaHsf18 (A4a)*, *TaHsf20 (A4a)*, *TaHsf31 (A6b)* and *TaHsf32 (A6b)* were significantly increased, while levels of *TaHsf7 (A2b)*, *TaHsf8 (A2b)*, *TaHsf9 (A2b)*, *TaHsf26 (A5)* and *TaHsf50 (B4b)* were significantly decreased (*P* < 0.05, Fig. 9). The qRT-PCR results were highly consistent with that of RNA sequencing data, confirming that it is reasonable to use RNA sequencing data to evaluate the expression level of transcripts in wheat Cd-response.

## Discussion

A growing body of evidence shows that *Hsfs* play essential roles in plant developmental and defense processes [16, 32–35]. Due to growing numbers of quality genomes available, putative functions of *Hsf* family genes have been predicted in many species, from the model plants *Arabidopsis* [13], rice [5] and maize [36], now to other crops, such as apple [21], Chinese cabbage [37], Chinese white pear [38] and pepper [39]. However, despite the global impact of wheat, as well as the importance of environmental Cd contamination, there has been limited investigation into the molecular basis of Cd accumulation, and the *Hsf* family in wheat.

Here we took advantage of the high quality wheat reference genome, to first identify 78 *Hsf* wheat genes and to characterize these bioinformatically (Table 1). A first contrast lies on the sheer quantity of these genes in wheat: while we identify 78 in wheat, there are only 21 *Hsfs* in Arabidopsis, 25 in rice, 30 in maize, 29 in Chinese white pear and 25 in apple [5, 13, 36, 38]. The vast majority of *Hsfs* can be categorized into three classes: A, B and C. The quantity of class A in Arabidopsis, rice, maize, Chinese white pear and apple are 15, 13, 16, 19 and 16, respectively. Class B *Hsfs* amount to 5, 8, 9, 8 and 7, in the five plants respectively. Finally, class C is represented by 1, 9, 4, 2 and 2, respectively. In contrast, of 78 putative wheat *Hsf* genes, 38 belonged to class A, 16 to class B and 24 to class C. Thus class C is relatively expanded in wheat in contrast to these other genomes.

We next investigated occurrences of possible gene duplication, which contributes differentially to the extension of specific gene families in plant genomes, and results from polyploidization or tandem and segmental duplication related [30, 40, 41]. In wheat, we found that homologous genes are gathered in group 3, 4 and 5 chromosomes, which was in line with the above analysis of chromosome localization. These results indicated that compared to other homologous chromosome groups, group 3, 4 and 5 chromosomes suffered less sequence loss and interaction impact. Three duplication events with the same chromosome and 14 segmental duplication events between various chromosomes were identified, suggesting that in wheat genome, the duplication events could play important roles in the extension of the *Hsf* cascade genes. A previous study indicated that more than 90% of the enhancement in regulatory genes in the Arabidopsis lineage were facilitated via genome duplications [42]. Compared with tandem duplications, segmental *Hsf* gene duplications were more often. This situation appeared in *Arabidopsis*, *maize*, *poplar* [21, 22, 36], and also in *wheat*.

Our phylogenetic analysis indicated that compared with *Arabidopsis*, *maize* and *rice*, *brachypodium Hsfs* were nearer to *wheat Hsf* proteins, which was in line with broader classifications. Identification of *Hsf* genes in *wheat* and its diploid ancestors, *T. urartu* and *A. tauschii*, which suggesting that the number of Hsf in a known subgenome was increased in transition from diploidy to hexaploidy (for A subgenome, 16 to 25 genes, and for D subgenome, 15 to 23 genes). These results further indicate that gene gain happened broadly during the formation of hexaploid [27].

Moreover, protein-protein regulatory interactions were constructed to provide inference of mechanisms of life activities and to explore potential biological functions for unknown proteins. Results showed that *TaHsf18 (A4a)* interacts with 24 wheat genes, including *ZAT6*, *STZ* and *S6K2*, suggesting it was mainly engaged in metal ion binding, intracellular signal transduction, and the negative regulation of cell proliferation. A previous study indicated that *ZAT6* coordinately activates the expression of phytochelatin synthesis-related gene and positively modulate Cd accumulation and tolerance by directly targeting GSH1 in Arabidopsis [43]. *HsfA4a* was also engaged in cadmium tolerance in wheat [19], suggesting it might be involved in metal ion binding via interacting with *ZAT6* to further play a role in cadmium tolerance in wheat. *TaHsf50* (B4b) interacts with 88 wheat genes, including *MYB15*, *MYB70*, *ZFP2*, *FMA*, and *HB31*, suggesting it is involved in regulation of transcription, regulation of jasmonic acid, metal ion binding and DNA binding. It has been reported that *MYB15* is required for the defense-induced synthesis of G-rich lignin and the constitutive synthesis of the coumarin metabolite scopoletin, both of which contribute to disease resistance against a hemibiotrophic bacterial pathogen [44]. *TaHsf44* (*B2c*) was found to interact with 30 wheat genes including *AGC2–1*, *WRKY39*, *BAG6* and *NF-YC2*, suggesting it is engaged in defense response, calmodulin binding, response to heat and flower development. *AtBAG6* can induce programmed cell death in yeast and plants [45]. Aspartyl protease-mediated cleavage of BAG6 plays an important role in autophagy and fungal resistance in plants [46]. GO analysis showed that 15 *TaHsfs* interacted genes were significantly enriched for transcription, DNA-templating, response to heat, transcription factor activity, sequence-specific DNA binding and calmodulin binding. It has been reported that *Hsf* family has a unique role as master modulators of thermotolerance, and were essential for plants survival under serious heat stress [9, 47].

Furthermore, we characterize *wheat Hsf* genes that expression throughout tissues and development stages. Many of these genes were highly expressed across development. For example, *TaHsf2, 3, 20, 17* and *45* were high expressed in roots, stems, leaves, spikes and grains including whole endosperm, starchy endosperm, transfer cells and aleurone layer, as well as seed coats during different developmental stages. It has been reported that Hsfs were involved in plant growth and development [9, 16]. Our results further indicated that *Hsf* genes play important regulatory roles in wheat growth, development and reproductive processes.

In addition, we comprehensively analyzed the expression levels of *Hsf* genes in response to drought, heat and Cd stresses to predict potential roles. The expression of most *Hsf* genes were differentially regulated in response to a given stress, which strongly suggests that they may be vital stress response genes. A previous study indicated that *Hsfs* are involved in responses to the abiotic stress as heat, cold, salt, drought and cadmium [13, 17, 19]. Our results first comprehensively illustrate that *Hsf* genes likely play important regulatory roles in wheat Cd stress response. Therefore, these genes stand as strong functional candidates for followup research into Cd stress in wheat.

## Conclusion

We present the first comprehensive identification and characterization of the wheat *Hsf* gene family. Through the latest available wheat genome information, total 78 putative wheat *Hsf* gens were identified through a genome-wide search, and categorized into class A, B and C subfamilies based on conserved motifs. Chromosome localizations, gene structures, conserved protein motifs, and phylogenetic relationship of these *TaHsfs* were comprehensively analyzed and strongly supported these classifications. Moreover, the gene duplication and homologous genes between wheat A, B and D sub-genome were also surveyed. Expression profiles of these *TaHsfs* through development and under various abiotic stresses were surveyed and provide strong functional candidates for followup work. Finally, through qRT-PCR analysis, 13 *TaHsf* genes were selected to verify their expression level in wheat under Cd stress, which provide top candidates for further functional analysis of *Hsf* genes in response to wheat Cd stress.

## Methods

### Identification and classification of *Hsf* gene family in wheat

The *Hsf* gene family was identified following the method as described by Wang et al. with some modifications [48]. First, to construct a local protein database, all the wheat (*T. aestivum L.*) protein sequences available were downloaded from the Ensemble database (http://plants.ensembl.org/index.html). Then, the database were searched with 100 known *Hsf* gene sequences collected from *A. thaliana* (21)*, O. sativa* (25)*, B. distachyon* (24) and *Z. mays* (30) using the local BLASTP program with an e-value of le-5 and identity of 50% as the threshold. Moreover, a self-blast of these sequences was performed to remove redundancy, the physical localizations of all candidate *Hsf* genes were checked and redundant sequences with the same chromosome location were rejected. Furthermore, all obtained *Hsf* protein sequences were analyzed to detect DBD domains and coiled-coil structures by the SMART and MARCOIL programs (SMART: http://smart.embl-heidelberg.de/, MARCOIL: http://toolkit.tuebingen.mpg.de/marcoil). Those protein sequences lacking the DBD domain or a coiled-coil structure were removed. Finally, to verify the existence of all the obtained sequences, BLASTN similarity searches against the wheat ESTs deposited in the NCBI database were performed. The theorectical pI (isoelectric point) and Mw (molecular weight) of the putative Hsf from *T. aestivum L* were calculated using compute pI/Mw tool online (http://web.expasy.org/compute_pi/), respectively. Classification of the three different groups A, B and C was based on structural characteristics and phylogenetic comparisons [49, 50].

### Gene structure construction, protein domain and motif analysis

Gene structure information were obtained from the Ensemble plants database (http://plants.ensembl.org/index.html). Conserved domains annotation was performed using Pfam (http://pfam.xfam.org/search), SMART (http://smart.embl-heidelberg.de/) and Heatster online tools [39]. All full-length amino acid sequences of the TaHsfs were used to identify conserved domain motifs by the Multiple Em for Motif Elicitation (MEME) tool [51]. The parameters were set as follows: maximum numbers of different motifs, 30; minimum motif width, 4; maximum motif width, 50.

### Chromosomal locations and gene duplication

Genes were mapped onto chromosomes by identifying their chromosomal position provided in the wheat genome database. Gene duplication events of Hsf genes in wheat were investigated based on the following three criteria: (a) the alignment covered > 80% of the longer gene; (b) the aligned region had an identity > 80% [52]. In order to visualize the duplicated regions in the *T. aestivum* genome, lines were drawn between matching genes using Circos-0.67 program (http://circos.ca/).

### Phylogenetic analysis

The N-terminal *Hsf* protein sequences containing the DBD and HR-A/B regions and parts of the linker between these two regions from *A. thaliana, O. sativa, B. distachyon, Z. mays* and *T. aestivum L.* were performed for multiple alignments by CLUSTALW and the results of alignment were used to construct phylogenetic tree using the NJ method in MEGA (version 6.0) [53]. Bootstrap test method was adopted and the replicate was set to 1000.

### Analysis of the *TaHsf* family orthologs in *T. urartu* and *A. tauschii*

The wheat- *T. aestivum*, wheat-*T. urartu* and wheat-*A .tauschii Hsf* genes were used to construct phylogenetic trees using neighbor-joining method with 1000 bootstrap replicates. According to these orthologous *Hsf* genes, a collinear map of the *T. urartu-*wheat A genome and *A. tauschii-*wheat B genome was created using genome visualization tool CIRCOS according to these orthologous *Hsf* genes. The locations of *Hsf* orthologous genes on the chromosomes of *T. urartu* and *A. tauschii* were obtained from the database published by Ling et al. [23] and Jia et al. [54], respectively.

### Network interaction analysis

The interaction network involving the *TaHsf* genes was based on the orthologous genes between Wheat and Arabidopsis using the AraNet V2 tool (http://www.inetbio.org/aranet/) [48]. Enrichment analysis was implemented by BiNGO, a cytoscape plugin, for gene ontology analysis and identifying processes and pathways of specific gene sets. Over-represented GO full categories were identified with a significance threshold of 0.01.

### The *TaHsf* gene expression analysis by RNA-seq data

To study the expression of *TaHsf* genes in different organs and response to stress, the wheat expression database (http://wheat.pw.usda.gov/WheatExp/) was used The FPKM (fragments per kilobase of transcript per million fragments mapped) value was calculated for each *Hsf* gene, the log2 transformed values of the *TaHsf* genes were used for heat map generation. *P*-values < 0.05 were taken as statistically significant thresholds [55].

### Plant materials, growth conditions, and treatments

The plant of wheat cultivar Chuanyu17, a high-Cd-accumulating cultivar, was planted in growth chambers at 23 ± 1 °C with a photoperiod of 16 h light/8 h dark. One-week-old seedlings were treated with 0 (H17CK) and 100 μM CdCl_2_ for 24 h (H17Cd). Roots from the plants with similar size were harvested separately and washed three times with deionized water. All the plant samples from three biological replicates were frozen in liquid nitrogen immediately and stored at − 80 °C for RNA extraction.

### RNA extraction and real-time quantitative RT-PCR (qRT-PCR) analysis

Total RNA was extracted from roots of Chuanyu17 in H17CK and H17Cd groups using TRIzol Reagent (Invitrogen, USA) according to the manufacturer’s instructions. RNA was quantified by using NanoDrop-1000 and RNA integrity was checked by electrophoresis. First strand cDNA was synthesized using HiScript IIQ RT SuperMix (Vazyme, R223–1). The primers used in the qRT-PCR analyses are listed in Additional file 9. β-actin was used as an internal control. The qRT-PCR was carried out using QuantiFast® SYBR® Green PCR kit (Qiagen, 204,054) according to the manufacturer’s instructions. Each treatment was repeated three times. The expression levels were calculated from the 2^-ΔΔCt^ value [ΔΔCt = (CT _target/Cd_ - CT _actin/Cd_ - (CT _target/control_ - CT _actin/control_)] [45].

## Additional files

Additional file 1: **Figure S1.** Motifs identified by MEME tools in *Wheat Hsfs*. Thirty motifs (1–30) were identified and indicated by different color. Motif location and combined *p*-value were represented. Motif 9 was found in TaHsf5, 6, 9, 10, 11, 13, 17, 18, 20, 23, 27, 28, 30, 31, 32, 45, 46, 52, 56, 59, 60, 64, 65, 66, 68, 73 and 75 which was covered by other motifs. **Figure S2.** Heat map of the expression profiles of Ta*Hsf* genes in different grain layers and a developmental timecourse. Log2 transformed FPKM values were used to establish the heat map. The red or green colors stand for the higher or lower relative abundance of each transcript in each sample. *P*-value< 0.05 were regarded as statistically significant. DPA means days post-anthesis. **Figure S3.** Heat map of the expression profiles of *TaHsf* genes under drought and heat stress treatments. Log2 transformed FPKM values were used to create the heat map. The red or green colors indicate the higher or lower relative abundance or each transcript in each sample. *P*-value< 0.05 were regarded as statistically significant. (PDF 580 kb)

Additional file 2: Motif sequences identified by MEME tools. Motif numbers corresponded to the motifs in Additional file 1: Figure S1. (XLSX 10 kb)

Additional file 3: The homologous *TaHsf* genes in wheat A, B and D sub-genomes and the Duplicated genes pairs identified in *wheat (XLSX 11 kb)*

Additional file 4: The list of the putative *Hsf* genes for *A.tauschii* and *T.urartu (XLSX 11 kb)*

Additional file 5: Details of *TaHsfs* and corresponding orthologs Hsfs in *T.urartu* and *A.tauschii (XLSX 11 kb)*

Additional file 6: The detail of 15 *TaHsf* orthologous genes in *Arabidopis thaliana (XLSX 10 kb)*

Additional file 7: Detail information of Network of *TaHsf* with other genes (XLSX 40 kb)

Additional file 8: Expression profiles of *TaHsf* in *wheat* under Cd stress (XLSX 13 kb)

Additional file 9: The Primers for *TaHsfs*. (XLSX 10 kb)

## Data Availability

The dataset and materials presented in the investigation is available by request from the corresponding author.

## Abbreviations

AHA: Activator motifs
Cd: Cadmium
DBD: DNA binding domain
FPKM: Fragments per kilobase of transcript per million fragments mapped
GO: Gene ontology
HR-A/B: Oligomerizationn domain
*Hsf*: Heat shock transcription factor
HSPs: Heat shock proteins
MEME: Multiple Em for Motif Elicitation
MT: Mutant
NES: Nuclear export signal
NLS: Nuclear localization signal
qRT-PCR: real-time quantitative RT-PCR
