# Supplementary figures and images for: Genome-wide identification, phylogenetic and expression analysis of the heat shock transcription factor family in bread wheat (Triticum aestivum L.)
Source: BMC Genomics. 2019 Jun 18;20:505. doi: 10.1186/s12864-019-5876-x (PMC6580518; doi:10.1186/s12864-019-5876-x)

Figure S1

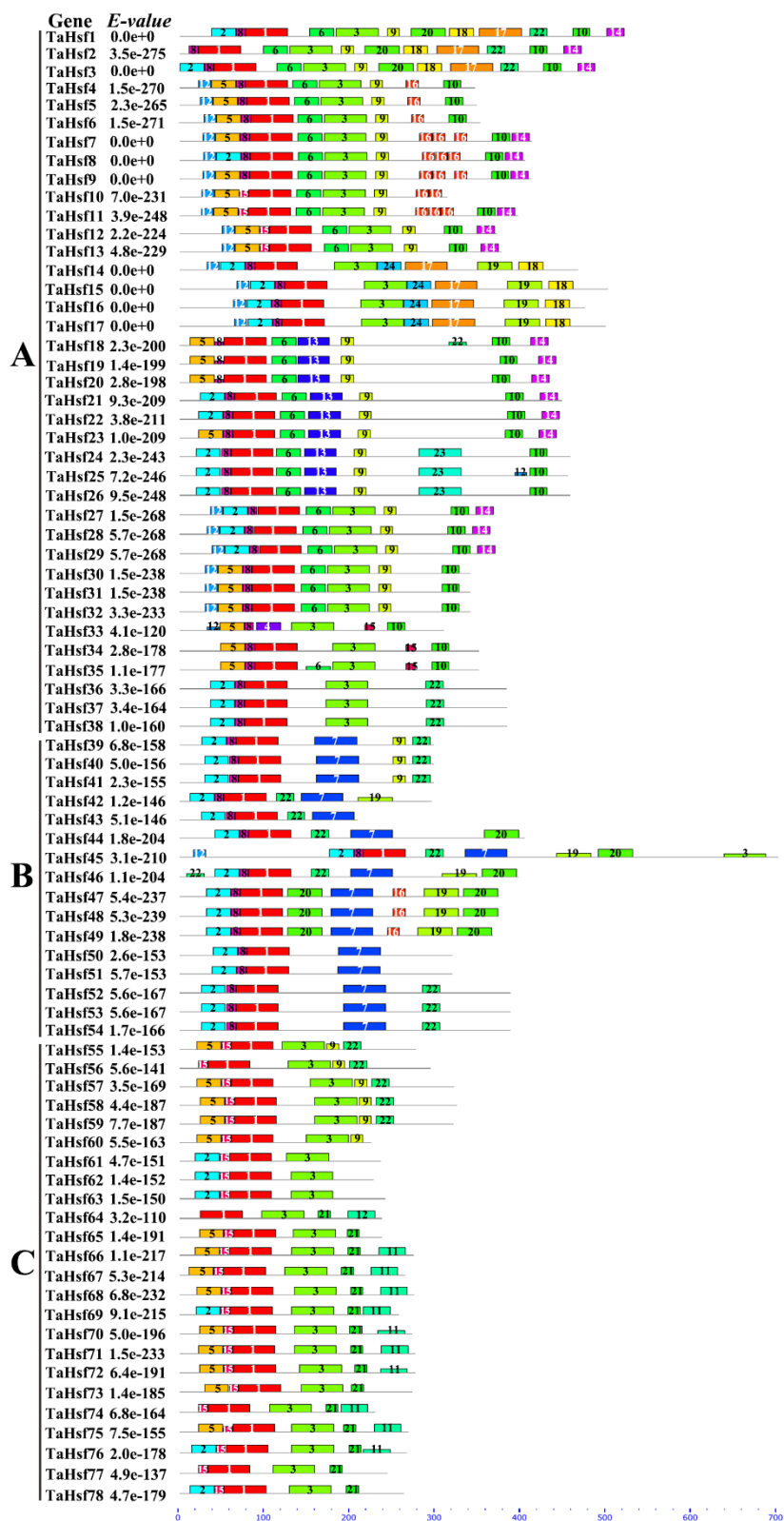

Figure S2

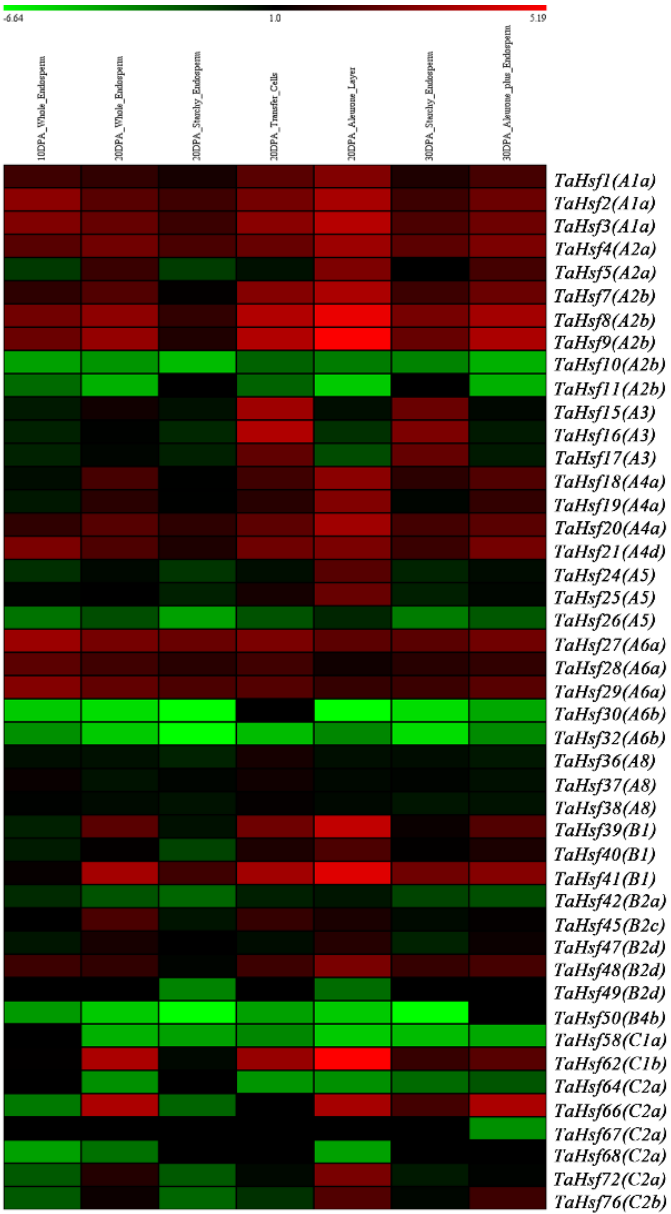

Figure S3

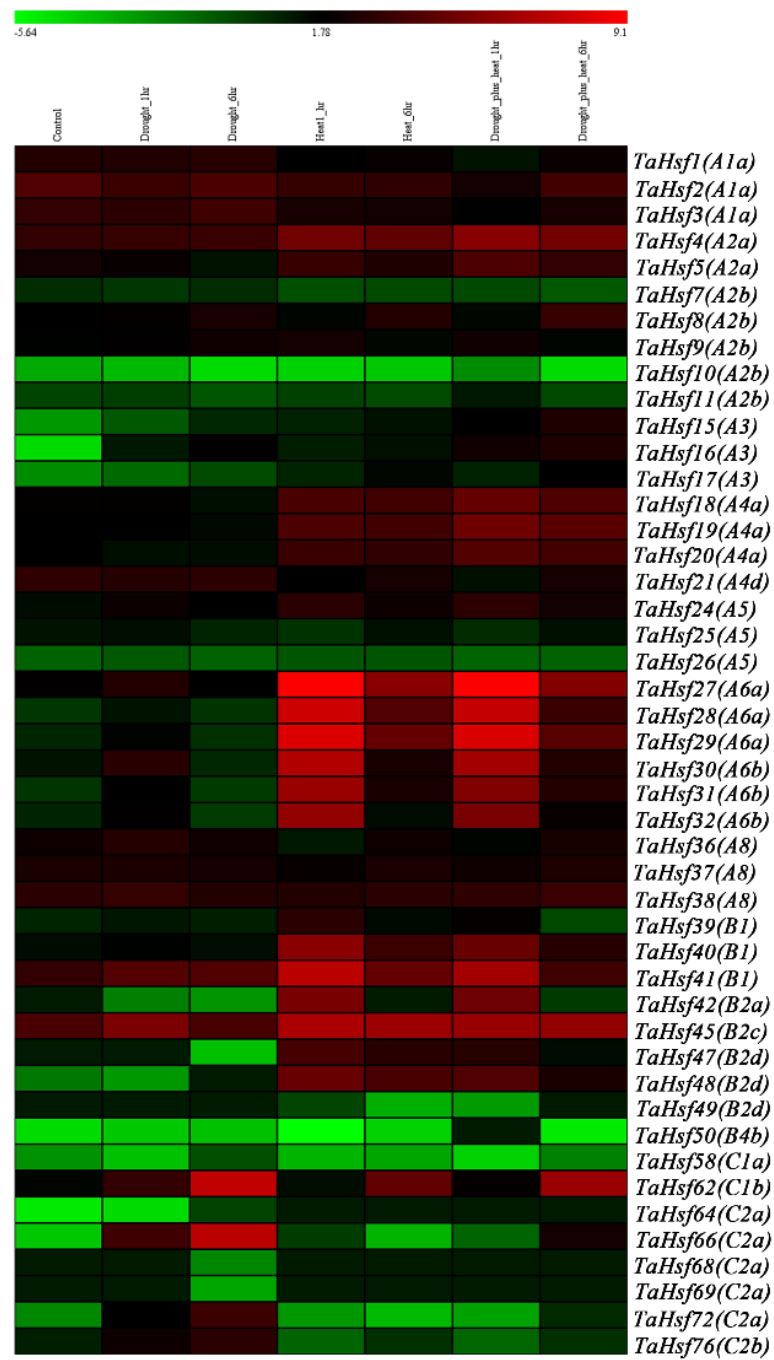

Supplement: Supplementary file 1 — Figure S1. Motifs identified by MEME tools in Wheat Hsfs. Thirty motifs (1–30) were identified and indicated by different color. Motif location and combined p-value were represented. Motif 9 was found in TaHsf5, 6, 9, 10, 11, 13, 17, 18, 20, 23, 27, 28, 30, 31, 32, 45, 46, 52, 56, 59, 60, 64, 65, 66, 68, 73 and 75 which was covered by other motifs. Figure S2. Heat map of the expression profiles of TaHsf genes in different grain layers and a developmental timecourse. Log2 transformed FPKM values were used to establish the heat map. The red or green colors stand for the higher or lower relative abundance of each transcript in each sample. P-value< 0.05 were regarded as statistically significant. DPA means days post-anthesis. Figure S3. Heat map of the expression profiles of TaHsf genes under drought and heat stress treatments. Log2 transformed FPKM values were used to create the heat map. The red or green colors indicate the higher or lower relative abundance or each transcript in each sample. P-value< 0.05 were regarded as statistically significant. (PDF 580 kb) [file 12864_2019_5876_MOESM1_ESM.pdf]
